# Clergy's Viewpoint Change Toward Mental Health and Stigma on Mental Illness: A Short Course Training

**DOI:** 10.3389/fpsyt.2022.864806

**Published:** 2022-04-01

**Authors:** Arsia Taghva, Ahmad Ali Noorbala, Mojgan Khademi, Alireza Shahriari, Mahdi Nasr Esfahani, Ali Asadi, Jafar Mohsenifar, Ali Yousefifard, Moussa Abolhassani, Jafar Bolhari, Ahmad Hajebi, Amir Mohsen Rahnejat, Haleh Shahed-haghghadam

**Affiliations:** ^1^Department of Psychiatry, Aja University of Medical Sciences, Tehran, Iran; ^2^Department of Psychiatry, Psychosomatic Medicine Research Center, Imam Khomeini Hospital, Tehran University of Medical Sciences, Tehran, Iran; ^3^Division of Child and Adolescent Psychiatry, Department of Psychiatry, School of Medicine, Imam Hossein Hospital, Shahid Beheshti University of Medical Sciences, Tehran, Iran; ^4^Department of Clinical Psychology, Aja University of Medical Sciences, Tehran, Iran; ^5^Department of Psychiatry, Iran University of Medical Sciences, Tehran, Iran; ^6^Deputy Mental Health Office, Social Addiction Ministry of Health, Tehran, Iran; ^7^Department of Psychology, Disaster and Trauma Research Center, 505 Hospital, Aja University of Medical Sciences, Tehran, Iran; ^8^Center for Services of Islamic Seminaries, Qom, Iran; ^9^Department of Epidemiology, School of Public Health, Iran University of Medical Sciences, Tehran, Iran; ^10^Department of Psychiatry, Spiritual Health Research Center, Iran University of Medical Sciences, Tehran, Iran; ^11^Department of Psychiatry, School of Medicine, Research Center for Addiction and Risky Behaviors, Social Injury Prevention Research Institute, Iran University of Medical Sciences, Tehran, Iran; ^12^Department of Clinical Psychology, Aja University of Medical Sciences, Tehran, Iran; ^13^Department of Clinical Psychology, Islamic Azad University, Varamin, Iran

**Keywords:** clergy, stigma, attitude, mental health, mental illness

## Abstract

**Background:**

As stigma is one of the main barriers in promoting the mental health, the present study was designed with the purpose of reviewing clergy's viewpoint regarding the effect of mental health workshops on these barriers.

**Methods:**

For this study, by order of Iran's Health Ministry, a questionnaire was designed to examine the clergy's viewpoint related to mental illnesses and the consequent stigma. Ten faculty members and psychiatrists confirmed the questionnaire's validity after some modifications. In this research, 30 members of the clergy from the main religious city in Iran's “Qom” Seminary attended the training workshops for 2 days. The data obtained from the clergy's responses were analyzed using the SPSS software (ver.16) and descriptive and analytical tests. Also, the significance level was considered *p* < 0.05 in all tests. The results exhibited that the mean and standard deviation (Mean ± SD) of the clergy's attitude domain and awareness before the workshop was 1.90 ± 26.30 and 8.31 ± 1.64, respectively. Also, average and standard deviation (Mean ± SD) of their attitude domain and awareness after the workshop was 1.95 ± 29.73 and 1.18 ± 10.70, respectively.

**Discussion:**

The present study, which was designed to examine the clergy's viewpoint toward mental illnesses and the consequent stigma in the most considerable religious base in the country, illustrated that one strategy for reducing mental illness stigma in religious communities can be by holding training sessions to promote the clergy's awareness of and attitude toward mental health.

**Conclusion:**

There was a significant statistical difference between their awareness and attitude scores before and after the workshop (*p* < 0.01). In the present research, the awareness and attitude of clergy toward mental health and stigma due to mental illness was relatively good and significantly increased by holding the workshop.

## Introduction

Attitude includes a set of beliefs, emotions, and behavioral intentions toward an object, person, or event. In other words, it is a relatively stable tendency toward a person, an object or an event that appears in feelings and behaviors (1). Unlike many studies conducted on the awareness and attitude of different groups of people toward the mental illness stigma in the western countries, few studies have been carried out in these areas of non-western countries (2, 3).

Stigma is specified as a negative stereotypical view associated with dogmatic beliefs and discrimination, which causes job, livelihood, and communication losses for the patients and those around them (4, 5). Stigma keeps the patients from social position and human reverence. These negative attitudes can be located in many countries (6–9). For example, in Nigeria, people have many mis-conceptions about mental patients; they are considered dangerous, unpredictable, rebellious, and unhelpful people. Other studies also underline these findings (1).

Unfortunately, mental patients receive this stigma from different sources, such as the community, family members, traditional and religious believers, and even mental health caregivers (10, 11). Negative opinions about mental illnesses can be observed throughout a community; and it seems that they come from culture and are fortified and continued by folklore and the media (12, 13). Prejudices related to mental health and those suffering from mental illness often are associated with unrealistic expectations. These unrealistic expectations provide conditions for the patients so that they internalize the stigma and, in turn, becomesa factor to stigmatize themselves; this state is known as self-stigmatization (14).

Previous studies have shown all aspects of psychiatry in Iran are affected by stigma; and in our society most mental health patients and their families suffer from stigma. Stigma in our society is a factor that prevents people with mental disorders from seeking treatment (2). Because of the culture of perfectionism in Iran that leads to stigma, there is secrecy in revealing statistics. The tendency of some authorities to hide mental illness and hide statistics is the result of cultural characteristics. In the current study, many participants mentioned cultural weaknesses as obstacles to reducing stigma. Moreover, media as a cultural representation of society does not have sufficient knowledge about mental health, which creates a negative image of mental health. Another qualitative study in Iran showed that about one third of families tried to hide their disorder from others (15, 16).

Different studies have illustrated that clergy are traditionally a haven for some people against the mental sufferings (17, 18). A study on Muslim Americans highlighted the role and importance of clergy in this regard (18). It is shown in another study that providing social services for depression treatment would be more effective and successful in the presence of church clergy (19). For drug-dependent in America, church is one of the most important sources of visiting and asking help from families and patients (20).

Numerous studies in the military have affirmed the role and influence of clergy in reducing mental illness stigma, reducing the sense of shame and guilt, and encouraging patients to meet with mental health practitioners (21–23). However, several studies have reported some problems, including clergy's lack of awareness of professional referral centers (18), linking non-related illness factors such as weakness in personality or undesirable topics for illness or the aggravation of mental illness stigma. Despite the negative attitudes in some clergy, who sometimes do not tend to refer the patients (23), recent studies have highlighted the role and importance of the presence of clergy in improving mental health in patients at different levels of prevention and treatment (24).

Moreover, many clergies are interested to take part in the promotion of mental health. In a study in which 65 clergy participated, 81% of them claimed that they required more training on depression and were willing to get more information from referral centers. The results indicated that the presence of clergy has a remarkable role in the reduction of mental illness stigma. In addition, their presence leads to early visits of patients (25), so holding joint seminars with psychiatrists, clergy, and church monks have been planned. Creating a fund to support for mental patients and helping to create housing for schizophrenic patients were among the advances of this initiation in Poland (26).

In an extensive qualitative study performed in Iran, lack of awareness has been proposed as one of the most important barriers to mental health promotion from beneficiaries (27). Numeral solutions have been suggested to tackle this problem named as increasing the awareness of influential groups on society such doctors and clergy (2).

From the perspective of Islam and teachings of the Prophet Mohammad, mental illnesses are divine providence and are separated from sin (18, 27) or divine torment, so this view about mental patients should be divulged among people by spiritual leaders and clergy as this is very effective in decreasing the stigma of mental illnesses in the society. Therefore, the present study was designed with the purpose of reviewing the clergy's viewpoint toward mental health and the stigma due to mental illnesses and also the effect of organizing workshops on changing their awareness and attitude in this area.

## Methods

After reviewing the related literature, examining the texts, and holding meetings with the experts, a questionnaire was designed to examine the clergy's viewpoints related to mental illnesses and the consequent stigma. To check the validity of the questionnaire, 10 faculty members and psychiatrists were selected; and they confirmed the questionnaire's validity after some modifications. Also, Cronbach's alpha calculation was used to define the reliability of the questionnaire and the total alpha coefficient for the questionnaire was calculated as 0.88.

At the beginning of the 1st day, the questionnaires were distributed among the clergy and the intended workshops were held after completing these questionnaires. At the end of the 2nd day, the questionnaires were returned back to the clergy to fill out them again.

The questionnaire included 15 questions, 11 related to awareness, and 4 to the attitude of clergy toward mental illness stigma, which were scored with the 5-point Likert scale. In the awareness domain, the minimum gained score was 0 and the maximum score was 33. Also, in attitude domain, the first gained score was 0 and the latter was 12. If higher scores were achieved, the awareness and attitudes of clergy would improve.

The workshop trainers were three faculty member psychiatrists and one psychologist, each having an experience of 15–25 years of mental health training on their resume, with two of professors collaborating with the Ministry of Health.

Regarding the workshops' training program, three other psychiatric professors, who were identified through the Qom Seminary Service Center, were also consulted. This led to minor changes to the workshop during the two consecutive sessions of the final program and after collaboration with the professors.

The data collected from the clergy's responses were analyzed using the SPSS software (ver.16), descriptive and analytical tests. The significance level was considered <0.05 in all tests.

## Results

In this study, 30 clergies from Qom Seminary attended 2-day training workshops. The clergy were invited to participate in the workshop following an invitation from the Qom Seminary Services Center. Thirty questionnaires were completed on the 1st day, 30 questionnaires were completed after the training, and four questionnaires were incomplete and excluded from the study.

The participating clergy's average age was 35.73 ± 3.26 years. All but one of the participants were married and five of them were female. The participants had completed the basic and higher Islamic seminary education (Table 1).

**Table 1:** Demographic variables of the clergy participating in the workshop.

**Variable**		**No. (%)**
Gender	Female	5 (20)
	Male	25 (80)
Marital Status	Single	1 (3.3)
	Married	29 (96.7)

The results exhibited that the attitude domain and awareness before the workshop was 26.30 ± 1.90 and 8.31 ± 1.64, respectively. Also, Mean ± SD of attitude domain and awareness after the workshop were 29.73 ± 1.95 and 10.70 ± 1.18, respectively. The data analysis indicated a significant statistical difference between clergy's attitude and awareness scores before and after organizing the workshop (*p* < 0.01) (Table 2).

**Table 2:** Mean and standard deviation (M ± SD) of the clergy's awareness and attitude pre- and post-workshop.

	**Awareness score (M ±SD)**	**Attitude score (M ±SD)**
Pre workshop	8.31 ± 1.64	26.30 ± 1.90
Post workshop	10.70 ± 1.18	29.73 ± 1.95
*P*-value	<0.001	<0.001

Pearson's coefficient test revealed that there was a significant relation between awareness and attitude score before and after holding the workshop (Table 3).

**Table 3:** Correlation between awareness and attitude score pre- and post-workshop.

	**Awareness**		
		***R***	***P*-value**
Attitude	Pre-workshop	0.566	0.001
	Post-workshop	0.426	0.030

## Discussion

The present study was conducted in Qom City, as the core of Shiite Muslim community in Iran and Eastern Mediterranean Region. In some countries, clergy have a remarkable role in interacting with the community, based on some studies on improving mental health (28). The present study, which was designed to examine the clergy's viewpoint toward the mental illnesses and the consequent stigma in the most considerable religious base in the country, illustrated that one strategy for reducing mental illness stigma in religious communities can be holding training sessions provided for promoting the clergy's awareness and attitude of mental health. Previous studies have exhibited a long background of mutual distrust between mental health scholars and religious clergy (22), however, it seems that such viewpoints have changed slightly. A study in America, following a training workshop held by religious clergy for psychiatric assistants, focused on the importance of collaboration with clergy and the important role of religion in enhancing mental health in the community (29). A study performed on older patients in 2005, highlighted the role of clergy in reducing mental illness stigma (30).

After a school shooting in Newtown, CT in America, a study investigated the clergy's performance in sermons. During the examination of the sermons, it was considered that clergy emphasized the importance of social and emotional support and funding the mental patient costs (31). It was determined in another study that working services for depression treatment would be more prosperous and forceful in the presence of a church clergy (19).

What makes this collaboration more effective and productive is to achieve a common language and more of a mutual understanding from mental health scholars after training, along with the acceptance of more scientific awareness by religion professionals. Research has portrayed that many clergy, despite their desire to help mental health professionals, had not achieved an agreeable outcome due to their lack of scientific background (18, 24, 32).

Some clergy lack acquired awareness or do not have willingness in referring patients to professional centers (23). But if clergy consider themselves as part of a mental health team member with doctors, nurses, and members of the treatment team, this confidence and participation may be increased (28). In many cases, the first referral of people suffering from mental disorders was to Christian (17) and Muslim (18) clergy.

Stigma can lead to poor attention by decision makers and stakeholders regarding mentally ill individuals (33). Previous studies have shown that fear and distrust from people suffering from mental disorders in society would be lessened with a better understanding of mental disorders among the general public (21). Our findings can promote the affairs of health policy makers in providing mental health education programs and community mental health services exploiting religious clergy and preachers.

Limitation of this current research include that clergy's skills, mental health education, and practice of clergy was not evaluated. Therefore, future studies should evaluate effectiveness of this course for the community and mentally ill individuals.

## Conclusion

In the present research, the awareness and attitude of clergy toward mental health and stigma due to mental illness was relatively good and significantly increased by holding the workshop. There was a significant statistical difference between their awareness and attitude scores before and after the workshop (*p* < 0.01).

## Data Availability Statement

The raw data supporting the conclusions of this article will be made available by the authors, without undue reservation.

## Author Contributions

AT, AN, MN, and MK contributed to conception and design of the study. AS and AA organized the database. AY and MA performed the statistical analysis. JB wrote the first draft of the manuscript. AH, AR, and HS-h wrote sections of the manuscript. All authors contributed to manuscript revision, read, and approved the submitted version.

## Funding

This study was conducted due to a grant from the Ministry of Health.

## Conflict of Interest

The authors declare that the research was conducted in the absence of any commercial or financial relationships that could be construed as a potential conflict of interest.

## Publisher's Note

All claims expressed in this article are solely those of the authors and do not necessarily represent those of their affiliated organizations, or those of the publisher, the editors and the reviewers. Any product that may be evaluated in this article, or claim that may be made by its manufacturer, is not guaranteed or endorsed by the publisher.
